# Predicting therapeutic efficacy of oral rehydration salts in children with vasovagal syncope

**DOI:** 10.3389/fped.2023.1164304

**Published:** 2023-04-13

**Authors:** Xiaojuan Du, Chunyan Tao, Xueying Li, Junbao Du, Ying Liao, Hongfang Jin

**Affiliations:** ^1^ Department of Pediatrics, Peking University First Hospital, Beijing, China; ^2^ Department of Statistics, Peking University First Hospital, Beijing, China; ^3^ State Key Laboratory of Vascular Homeostasis and Remodeling, Peking University, Beijing, China

**Keywords:** oral rehydration salts, nomogram, vasovagal syncope, predictive model, body mass index, hematocrit

## Abstract

**Objective:**

This study was designed to develop an easy-to-perform and inexpensive measure to predict efficacy of the oral rehydration salts (ORS) in children with vasovagal syncope (VVS).

**Materials and methods:**

Children diagnosed with VVS and treated with ORS for a median of 3 months at the Peking University First Hospital, China, were enrolled and followed up. Demographic data, clinical hemodynamic parameters, and variables related to red blood cells were collected at the baseline. On the basis of changes in symptom scores after treatment, participants were divided into effective or ineffective groups at the end of the follow-up. Logistic regression analysis was used to investigate parameters related to therapeutic efficacy of ORS and a predictive model of ORS effectiveness was created. The predictive efficiency was evaluated using the receiver operating characteristic curve. The accuracy/consistency was evaluated by the Hosmer–Lemeshow test and calibration curve. Internal validation was done using the bootstrap approach.

**Results:**

Totally 97 pediatric participants were included in the study and 4 (4.1%) were lost during the follow-up. ORS therapy was effective in 46 children and ineffective in 47 children. Children in the effective group had higher baseline red blood cell count, hemoglobin, and hematocrit than those in the ineffective group (*p* < 0.01). Through logistic regression analysis, the baseline hematocrit and body mass index (BMI) were included in predictive model for the response to ORS treatment. The predictive efficacy of the model showed an area under the curve of 0.77 (*p* < 0.01). The predicted probability cut-off value of 0.5 was found to be optimal, with a resulting sensitivity of 67.4% and specificity of 80.9%. In the Hosmer–Lemeshow test, *p*-value was 0.75, and the calibration plot showed a good model fitness. Internal validation was performed using the bootstrap approach (*n* = 1,000), showing 95% confidence interval of 0.67–0.86.

**Conclusion:**

Hemoglobin combined with BMI was useful for predicting the therapeutic efficacy of ORS in children with VVS.

## 1. Introduction

Syncope is defined as a temporary loss of consciousness caused by reversible cerebral hypoperfusion with heterogeneous etiology. The most prevalent underlying cause of syncope is vasovagal syncope (VVS), which accounts for approximately 60% of all the syncopal causes in children (1). The pathogenesis of VVS is complex and includes, but is not limited to, hypovolemia (2–4), enhanced sympathetic nerve activity (5, 6) and catecholamine levels (7), and excessive peripheral vasodilation (8). It was found that during an attack of VVS, the orthostatic stress, which was the major trigger, may initially stagnate blood in the veins of lower extremities due to gravitational forces (9) and thereby reduced the circulating blood volume, which activated an aberrant autonomic regulation (10), resulting in bradycardia (11, 12) and/or hypotension, and ultimately decreasing the cerebral blood flow (13–15) and oxygen saturation (15, 16). The relative central hypovolemia can be the initial factor that promotes the whole process.

In clinical practice, the efficacy of oral rehydration salts (ORS) therapy is not satisfactory for VVS (17, 18), although it is the treatment targeting hypovolemia. Considering the complex pathogenesis of VVS as mentioned above, we hypothesized that ORS would be an ideal therapeutic option for children suffering from VVS with hypovolemia as the main pathogenesis, other than VVS with the abnormal sympathetic nerve activity (5, 6) and excessive vascular dilation.

Therefore, to increase the effectiveness of ORS on VVS patients, it is extremely necessary to find out some stable and easy-to-perform predictors or develop practical predictive models to indicate those VVS children with hypovolemia as the main pathogenesis to select ORS as the clinical therapeutic options. Previously, body mass index (BMI) (19) or 24-hour urinary sodium excretion (20) were utilized to predict the effectiveness of ORS in patients with VVS. Although both can indirectly reflect blood volume, the predictive value of a single indicator is always limited and has its own limitations. BMI can be easily influenced by congenital factors such as genetic background (21) and acquired factors such as nutrition and economic conditions (22, 23). Twenty-four-hour urine is not convenient to collect for outpatients. Some previous studies have suggested that hematocrit and red blood cell-associated parameters, which are relatively stable and easy to obtain, can indicate blood volume (14, 24). In order to predict the effectiveness of ORS in children with VVS, we sought to develop an easy-to-perform prediction model in this study.

## 2. Materials and methods

### 2.1. Subjects

Inclusion criteria for the participants were (1) children hospitalized due to VVS between July 2012 and December 2022, as determined by the existing criteria (25); (2) age of the patients ranged from 5 to 17 years old; and (3) patients treated by health education (avoidance of triggers and physical counterpressure maneuvers) and ORS.

Exclusion criteria were (1) children with uncertain illness or other causes of transient loss of consciousness such as cardiogenic syncope, orthostatic hypotension, or psychogenic pseudosyncope; (2) patients with anemia or other hematological disorders; (3) irregular use of ORS; (4) children treated with other medicines, such as beta-blockers and hydrochloride midodrine; and (5) patients with incomplete medical data.

### 2.2. Therapy and follow-up

ORS was administered daily to all participants (Anjian Pharma Company, Xi’an, China). Each bag included 3,375 mg of waterless glucose, 725 mg of sodium citrate, 650 mg of sodium chloride, and 375 mg of potassium chloride (25, 26). The treatment with ORS was initiated from the diagnosis of VVS made after hospitalization, and the median duration of treatment was 3 months.

Three months after starting therapy, follow-up was conducted by a professional investigator by telephone or outpatient visits. Demographic information, symptoms and signs, and baseline hemodynamic and hemocytometric variables were collected before the therapy. As indicated before (19), symptom score was calculated before and at the end of the follow-up depending on the occurrence and frequency of syncope: 0 point indicated no syncopal event; 1 point, 1 event per month; 2 points, 2–4 events per month; 3 points, 2–7 events per week; 4 points, >once per day. When the symptom score decreased by at least 1 point, the treatment was considered to be effective (19).

### 2.3. Collection of demographic data, symptoms, and baseline hemodynamic and hemocytometric variables

Demographic information and symptoms were recorded. Using a Dash 2000 multichannel physiological monitor (General Electric Company, New York, United States), baseline hemodynamics was obtained.

Before collection of the blood samples, participants were requested to fast for 4 h, and then venous blood (2–3 ml) was collected in the morning. All the patients did not have symptoms like vomiting and/or diarrhea that might cause dehydration 3 days before the blood sample collection. Sysmex XE-5,000 (Sysmex Corporation, Kobe, Japan) was used to measure hemocytometric variables.

### 2.4. Head-up tilt test

The head-up tilt test (HUT) was conducted in a darkly lit, warm, quiet environment. Before HUT, patients must fast for 4 h and quit autonomic nerve-affecting medicines for 5 half-lives. Children were monitored for 20 min while supine on a tilt table (SHUT-100A, Standard, Jiangsu, and ST-711, Juchi, Beijing, China). The tilt table was slanted at 60° and the test was terminated when a positive reaction appeared, or 45 min-process was completed.

### 2.5. Statistical analysis

SPSS 26.0 (IBM Corp, Armonk, NY, United States) was used in the present study. The Kolmogorov–Smirnov test for normal distributivity was performed, continuous data were analyzed with the *t-*test and nonparametric Mann–Whitney *U* test, and categorical data were analyzed with the *χ*^2^ test. Variables with *p* less than 0.05 and demographic variables were included into model built by logistic regression (stepwise regression method). Multicollinearity between the variables was checked using their corresponding variance inflation factor (VIF). Evaluation of the model was done by the receiver operating characteristic (ROC) curve, calibration plot (calibrate, MASS package), Hosmer–Lemeshow test, and decision curve analysis (DCA) (R, version 4.2.0, rmda package). A bootstrap self-sampling method (*n* = 1,000) was used to internally validate the model. A nomogram was used to illustrate the model results. *p* < 0.05 indicated a significant difference.

## 3. Results

### 3.1. The baseline items

**Figure 1:**
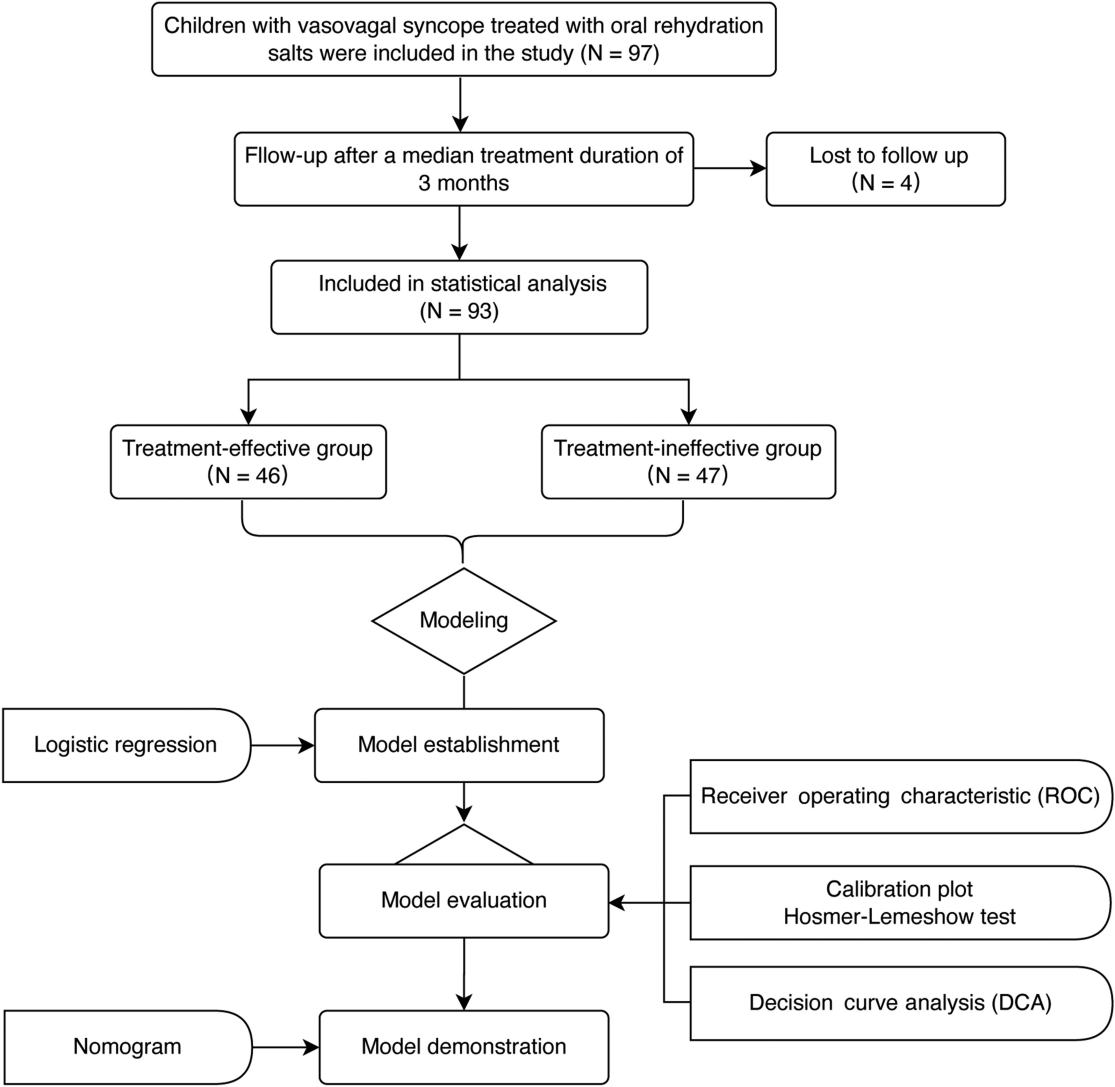
The flowchart of the research.

Totally 97 patients treated with ORS for a median of 3 months were enrolled; however, 4 were subsequently lost during follow-up (Figure 1). According to changes in symptom scores, the ORS therapy was effective in 46 cases (effective group) and ineffective in the other 47 cases (ineffective group). There was no significant difference in pretherapy symptom score, age, BMI, duration of therapy, and hemodynamic parameters between the two groups (*p* > 0.05). There was statistical difference in red blood cell count, hemoglobin, and hematocrit between the two groups (*p* < 0.05). The levels of red blood cell count, hemoglobin, and hematocrit were higher in the effective group than those in the ineffective group (*p* < 0.01, Table 1).

**Table 1:** Baseline demographic and clinical data between effective and ineffective groups.

Items	Ineffective, *N* = 47	Effective, *N* = 46	*p*-value
Age (years)^a^	10.00 (8.00–12.50)	12.00 (8.25–13.00)	0.195
Gender^b^			0.114
Male	14 (30%)	21 (46%)	
Female	33 (70%)	25 (54%)	
Pretherapy symptom score (points)^a^	1.00 (1.00–1.00)	1.00 (1.00–1.25)	0.285
Symptom score at the end of follow-up (points)^a^	1.00 (1.00–1.00)	0.00 (0.00–0.00)	<0.001
Duration of therapy (months)^a^	3.00 (2.00–3.00)	3.00 (2.00–3.00)	0.847
Body mass index (kg/m^2^)^a^	17.44 (15.69–19.38)	16.41 (15.50–19.15)	0.341
Pretherapy heart rate (bpm)^a^	76 (71–87)	75 (71–85)	0.744
Pretherapy systolic blood pressure (mmHg)^a^	105 (98–112)	104 (97–115)	0.881
Pretherapy diastolic blood pressure (mmHg)^c^	62 (8)	62 (8)	>0.9
Pretherapy red blood cell count (10^12^/L)^c^	4.66 (0.35)	4.90 (0.36)	0.002
Pretherapy hemoglobin (g/L)^c^	135 (9)	142 (10)	0.001
Pretherapy hematocrit (%)^c^	39.40 (2.66)	41.56 (2.49)	<0.001
Pretherapy mean corpuscular volume (fl)^a^	84.8 (82.4–86.7)	84.2 (82.2–88.3)	0.620
Pretherapy mean corpuscular hemoglobin (pg)^c^	28.86 (1.19)	28.97 (1.26)	0.666
Pretherapy mean corpuscular hemoglobin concentration (g/L)^a^	342 (336–348)	342 (334–347)	0.797
Pretherapy red blood cell distribution width (%)^c^	12.59 (0.58)	12.81 (0.67)	0.099

^a^ Median (interquartile range): *U* test.

^b^ *N* (%): *χ*^2^ test.

^c^ Mean (SD): *t*-test.

### 3.2. Construction of predictive models

The items with *p*-values below 0.05 (red blood cell count, hematocrit, and hemoglobin) and demographic variables (gender, age, and BMI) were tested for multicollinearity. According to multicollinearity analysis (Table 2, VIF < 10), the six items mentioned were included in multivariate regression analysis. As per the results of regression analysis, hematocrit and BMI were two independent factors associated with the efficacy (Table 3), and the regression equation is shown as

log⁡it(p)=−12.438−0.189×BMI+0.389×hematocrit

**Table 2:** The multicollinearity analysis.

Items	Tolerance	VIF
Red blood cell count (10^12^/L)	0.155	6.465
Hematocrit (%)	0.108	9.266
Hemoglobin (g/L)	0.139	7.173
Age (years)	0.561	1.783
Body mass index (kg/m^2^)	0.795	1.257
Gender	0.727	1.376

VIF, variance inflation factor.

**Table 3:** Multivariate logistic regression of the construction of predictive models.

Items	B	SE	Wald	OR	95% CI	*p*-value
Body mass index (kg/m^2^)	−0.189	0.094	4.046	0.827	0.688–0.995	0.044
Hematocrit (%)	0.389	0.104	13.933	1.475	1.203–1.809	<0.001
Constant	−12.438	3.987	9.734	—	—	0.002

CI, confidence interval; SE, standard error; OR, odds ratio.

### 3.3. Evaluation of predictive model

#### 3.3.1. Degree of differentiation

**Figure 2:**
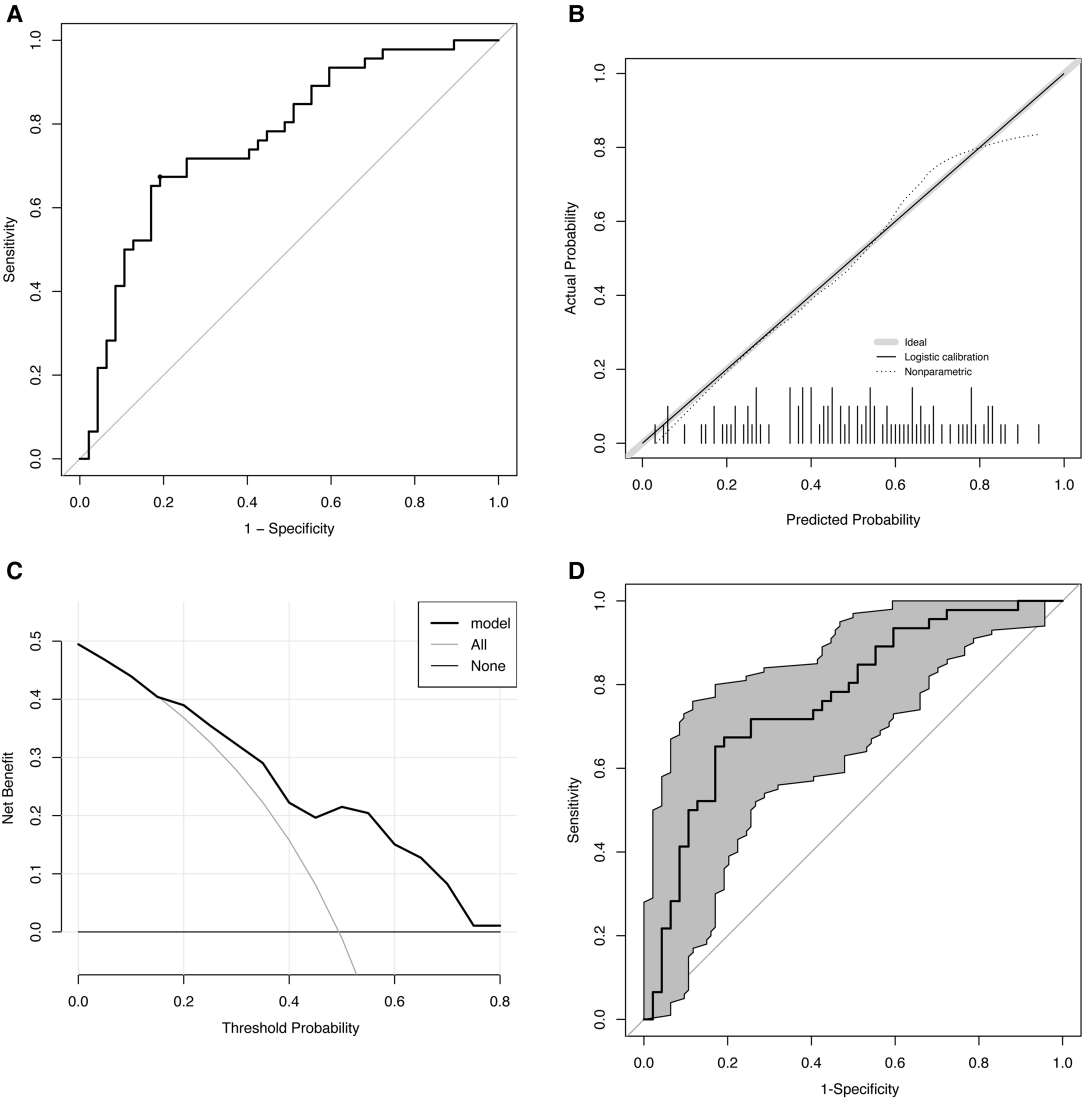
Evaluation of the predictive model. (**A**) ROC. AUC: 0.77 (*p* < 0.01), cut-off value: 0.5, sensitivity: 67.4%, and specificity: 80.9%. (**B**) Calibration curve. The gray line indicates the ideal reference line where predicted (x-axis) and observed probability (y-axis) coincide. The black line represents the performance of the model. The closer the black line is to the gray line, the more accurately the model predicts. (**C**) DCA. ORS treatment is regarded as an intervention, and the purpose of the ORS is naturally to obtain a “good result” (positive outcome). DCA includes results for “ORS for all,” “ORS for none,” and “ORS for model.” The x-axis displays the probability threshold. The y-axis indicates the degree of benefit to which the patient benefited from the intervention (ORS). (**D**) Bootstrap-corrected receiver operating characteristic curve (*n* = 1,000). The gray part is the 95% confidence interval (0.67–0.86). ROC, receiver operating characteristic curve; AUC, area under receiver operating characteristic curve; DCA, decision curve analysis; ORS, oral rehydration salts.

ROC analysis showed that the area under curve (AUC) was 0.77 (*p* < 0.01). To predict the effectiveness, the ideal cut-off value was 0.5, producing a sensitivity of 67.4% and a specificity of 80.9% (Figure 2A).

#### 3.3.2. Calibration

The Hosmer–Lemeshow test indicated that the predicted and observed values were not substantially different (*p* = 0.75), indicating a good model suitability. The calibration curve is shown in Figure 2B.

#### 3.3.3. Clinical utility

Clinical utility was analyzed using DCA, and the results are shown in Figure 2C. Our study showed that within 0.1–0.8 of the threshold probability, the net benefit of the prediction model ranged between 0.01 and 0.5. We found that treatment decisions based on model predictions will provide higher net benefit than that without using the model. For instance, at a threshold of 0.4, the calculated net benefit based on intervention for model is about 0.22, while the calculated net benefit based on intervention for all is about 0.16.

#### 3.3.4. Internal validation

Internal validation of the data performed using the bootstrap approach (*n* = 1,000) showed a good predictive accuracy: an AUCof 0.77 and 95% confidence interval (CI) of 0.67–0.86 (Figure 2D).

### 3.4. Nomogram of the predictive model

**Figure 3:**
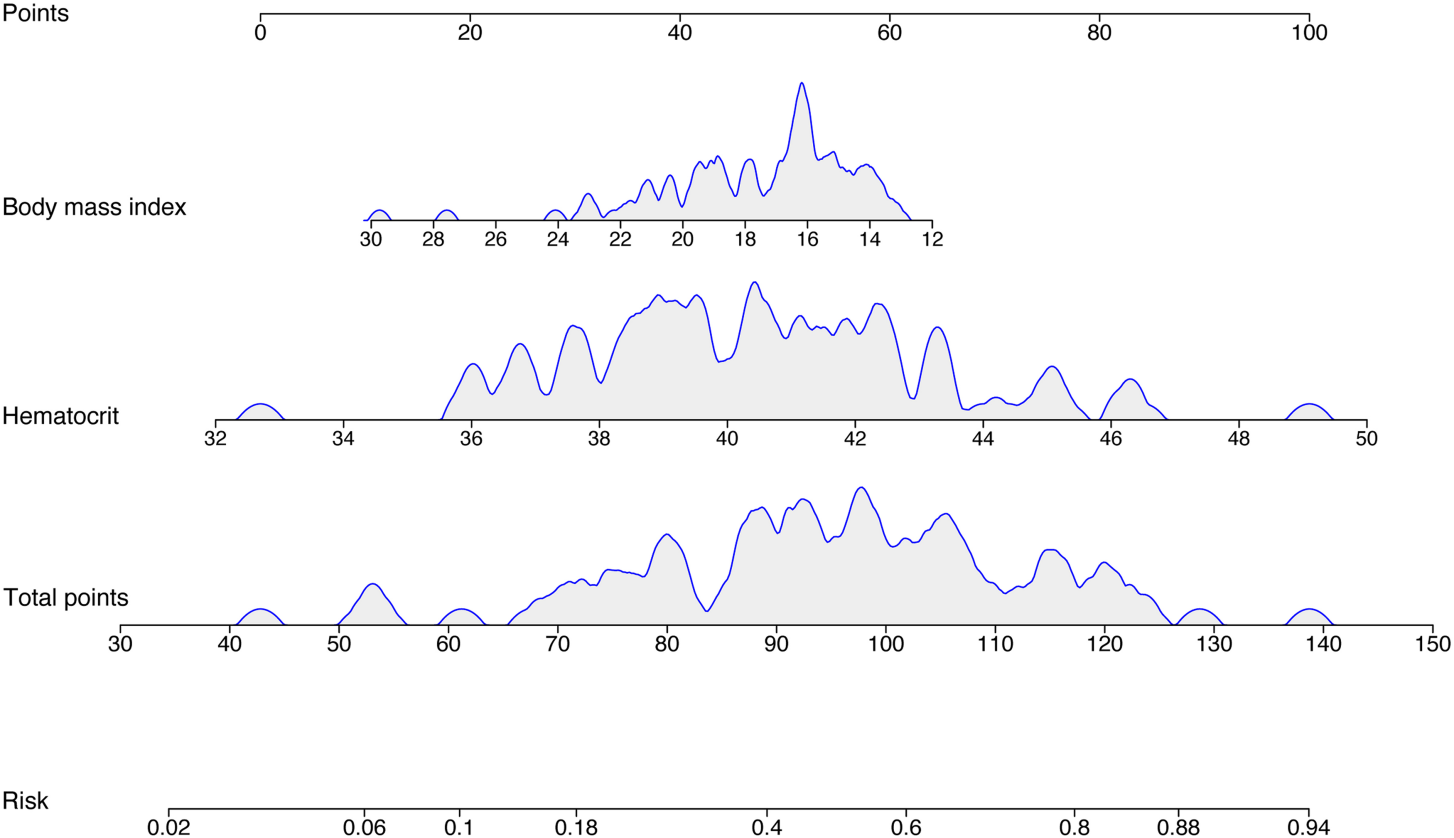
Nomogram of the prediction model. The nomogram is applied by placing each patient's items on a variable axis and then drawing a vertical line to determine the points for each item. The total points were then placed on the number axis of the total points line, and a vertical line is drawn to the final axis.

A nomogram of predictive model consisting of the two factors (hematocrit and BMI) were created to visually predict ORS treatment efficacy based on different scores (Figure 3). A nomogram is a graphical representation of the solution to an equation that approximates the risk with reasonable accuracy. For instance, when the BMI is 27 kg/m^2^, the score is approximately 20%. When hematocrit is 39%, the score is approximately 40%. The total score is approximately 60, resulting in a predicted effective rate of approximately 0.1. Therefore, ORS treatment is not recommended.

## 4. Discussion

We followed up 97 children with VVS treated with ORS, and after a median duration of 3 months of treatment, 46 patients responded well to the treatment, with an effective rate of less than 50%. There were statistical differences in gender, red blood cell count, hemoglobin, and hematocrit between effective and ineffective groups. Subsequently, logistic regression was carried out to generate a new model in which hematocrit and BMI were related to the effectiveness of ORS therapy. The model was evaluated, and the ROC curve demonstrated that the sensitivity and specificity were 67.4% and 80.9%, respectively, and the DCA suggested that using the model to predict the effectiveness of ORS in children with VVS would improve clinical outcomes. We also presented the results in a nomogram to facilitate the decision-making by clinicians and to enhance the practicability of the model.

To increase the water and salt intake is an acknowledged measure in the management of VVS. However, a confusing fact is that the empirically unselected use of oral rehydration salts as a first-line therapy for children with VVS showed limited efficacy (17). Therefore, there is a strong need to find out useful markers to indicate the VVS patients with hypovolemia as the main pathogenesis to guide the ORS therapy. It has been noted that BMI and 24-hour sodium excretion collection have correlation with blood volume (27) and were used to predict the effectiveness of ORS therapy for children with VVS (19, 20). However, as an indirect correlative factor to blood volume, BMI may be influenced by the genetic factors as well as the content of body fat, which may challenge the value of BMI in predicting the effectiveness of fluid and salt supplementation. Although the predictive power is similar between our model and 24-hour urinary sodium excretion based on the AUC of ROC curve (0.77 vs. 0.84), the parameters in our study are more readily available as the latter needs a 24-hour urine collection procedure. Furthermore, our model can provide an exact estimated effective rate, which is an important advantage of a nomogram. The nomogram of this study makes it more intuitive for clinical applications. In this study, we demonstrated that hematocrit combined with BMI was useful in the prediction of the efficacy of ORS treatment in children with VVS. Moreover, a clinical utility analysis was performed, and a graphic tool was created to assist the clinical decision-making, which may improve the current individualized treatment.

However, there are several limitations of our research. Due to the single-center design, selection bias was inevitable. In the future, multicenter-based studies are expected. The follow-up duration was not long enough, and the sample size was not large enough. We will continue to confirm the conclusions in future studies to achieve sufficient quantity. Second, the therapeutic efficacy was only evaluated by patients’ symptoms, while other indicators involving investigations such as negative rate of HUT need to be considered in future studies. Despite the limitations, our results suggest that hematocrit combined with BMI constitutes a clinically valuable model for predicting the efficacy of ORS therapy in pediatric patients with VVS, providing a useful strategy for the personalized treatment of VVS.

## 5. Conclusions

This study confirmed the hematocrit and BMI as useful, easy-to-obtain, and inexpensive indicators to forecast the therapeutic response to ORS in children with VVS, and we constructed a nomogram for an individualized prediction of the usefulness to ORS in pediatric VVS patients, which would greatly help in making treatment strategies.

## Data Availability

The raw data supporting the conclusions of this article will be made available by the authors, without undue reservation.
